# Environmentally Friendly and Simple Recycling of Titanium Alloy Scrap via Deoxygenation with Hybrid Hydrogen Plasma Arc

**DOI:** 10.1002/advs.202414747

**Published:** 2025-01-21

**Authors:** Botao Jiang, Liang Wang, Guotao Zhou, Xu Liu, Hongyu Lu, Guoqiang Zhu, Jiaxin Du, Chunzhi Zhao, Baoxian Su, Binbin Wang, Ruirun Chen, Yanqing Su

**Affiliations:** ^1^ School of Materials Science and Engineering Harbin Institute of Technology Harbin 150001 China; ^2^ Zhengzhou Research Institute Harbin Institute of Technology Zhengzhou 450000 China; ^3^ National Key Laboratory for Precision Hot Processing of Metals School of Materials Science and Engineering Harbin Institute of Technology Harbin 150001 China

**Keywords:** deoxygenation, hydrogen plasma, mechanical properties, microstructure, recycle, titanium scrap

## Abstract

The potential of hydrogen plasma arc technology for the efficient deoxygenation and recycling of titanium alloy scrap is explored. The results of thermodynamic analysis reveal that hydrogen plasma is suitable for oxygen removal. The intermediate stages of the deoxygenation process are sequentially analyzed, showing that the hydrogen plasma arc primarily facilitated the reduction and dissolution of oxides as well as eliminated interstitial oxygen. The experimental results demonstrate that the deoxygenation dynamics are governed by the interaction between the hydrogen partial pressure and the surface area of the hydrogen plasma arc irradiation range. Furthermore, the results of the detailed characterization of the recycled titanium alloys indicate that hydrogen plasma arc technology not only reduces the oxygen content to levels compliant with industrial standards for Ti‐6Al‐4 V but also substantially enhances the tensile strength and ductility of Ti‐6Al‐4 V compared with those of conventional cast titanium alloys. The results of microstructural analysis show that hydrogen tends to concentrate in the β phase, whereas oxygen is predominantly found in the α phase. These findings highlight the effectiveness of hydrogen plasma arc technology, which offers environmental benefits and improves the performance of the resulting material, indicating its promise as a solution for titanium recycling.

## Graphical abstract

The potential of hydrogen plasma technology for the recycling of titanium alloy scrap is investigated. Thermodynamic and experimental analyses demonstrate its efficacy in oxygen removal, yielding Ti‐6Al‐4V alloys with enhanced mechanical properties while ensuring compliance with industrial oxygen content standards. The study highlights the efficacy of this process in improving alloy performance while advancing environmental sustainability objectives.

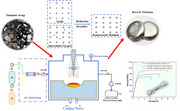

## 1 Introduction

Ti alloys are at the forefront of advanced materials and offer a suite of properties that are highly valued across a range of high‐tech industries. Their strength‐to‐weight ratio, corrosion resistance, and high‐temperature performance have led to their common use in the aerospace, biomedical, automotive, and other sectors.^[^ ^1^, ^2^, ^3^, ^4^, ^5^, ^6^, ^7^, ^8^, ^9^ ^]^

Machining is the preferred method for fabricating titanium alloys to avoid residual stresses and achieve precise tolerances in of high‐performance components, particularly those critical in aerospace and medical applications. However, machining is inherently material‐inefficient; with an average use rate of only 40%, machining generates substantial quantities of titanium alloy waste. Moreover, the industrial production and processing of metallic materials, including titanium, consume large amounts of energy consumption and emit greenhouse gases and pollutants.^[^ ^10^, ^11^, ^12^ ^]^ Hence, a method of recycling of titanium alloy resources must be developed to ensure the sustainable development of the titanium industry.^[^ ^13^ ^]^

A comprehensive titanium recycling system is not available, unlike for steel and aluminum. The high reactivity of titanium presents distinct challenges in its recycling. The two primary sources of recyclable titanium materials are the low‐grade sponge titanium produced during the Kroll process and the cutting waste generated during machining. Both scrap types are primarily contaminated with Fe and O. High‐density Fe impurities are effectively removed using melting and deposition methods. However, the key challenge facing titanium alloy recycling is the removal of oxygen due to the strong affinity between titanium and oxygen.^[^ ^14^, ^15^, ^16^, ^17^ ^]^ Therefore, the critical aspect in recycling titanium alloy scrap is the effective removal of oxygen.

The current titanium alloy scrap recycling methods, which are effective to varying degrees, have notable limitations. Traditional approaches, such as blending low‐oxygen sponge titanium with scrap, followed by remelting processes such as vacuum arc remelting (VAR), electron beam melting (EBM), or plasma arc melting (PAM), depend on the availability of high‐purity sponge titanium; these approaches are costly and energy‐intensive.^[^ ^10^, ^11^ ^]^ Electrochemical techniques, including molten salt electrolysis, have the potential to remove impurities but are less effective when the materials are contaminated with molten salts, and controlling titanium deposition is challenging. Newly developed methods, such as hydrogen‐assisted magnesiothermic reduction (HAMR), provide a cost‐effective route for deoxidation but face issues regarding scalability and industrial integration. Similarly, calcium deoxidation effectively reduces the oxygen content from titanium alloy scrap but is operationally complex due to the need to handle molten calcium and its byproducts.^[^ ^18^, ^19^, ^20^, ^21^, ^22^, ^23^, ^24^ ^]^

Using hydrogen plasma arc melting for deoxygenation has advantages such as a shorter process flow, higher deoxygenation efficiency, and a lower likelihood of secondary pollution compared with methods such as calcium, halide, and electrolytic deoxygenation. Hydrogen plasma arc melting offers a viable pathway for the recycling of titanium alloy scrap. This technology has been used for the smelting and preparation of metals. Raabe et al. studied the reduction of hematite, red mud, and iron oxides using a hydrogen‐argon plasma arc in an electric arc furnace. The iron conversion kinetics were found to be dependent on the balance between the initial hematite mass and arc power.^[^ ^25^, ^26^, ^27^, ^28^, ^29^, ^30^, ^31^ ^]^ Minura et al. used hydrogen‐argon plasma arc melting to purify commercial‐grade titanium, which further reduced the concentrations of impurities.^[^ ^32^, ^33^, ^34^ ^]^ However, research on the deoxygenation of titanium scrap using hydrogen plasma melting is limited, and the microstructure and mechanical properties of recycled titanium alloys have not been verified or tested. Based on previous studies on hydrogen‐induced modifications of titanium alloys,^[^ ^35^ ^]^ we think that the mechanical properties of titanium alloys recycled using this technology can be enhanced.^[^ ^36^, ^37^, ^38^, ^39^, ^40^ ^]^

In this study, we sequentially evaluated the deoxygenation process by employing hydrogen‐argon plasma arc melting to recycle titanium alloy scrap. Our investigation focused on the dynamic alterations in the composition, microstructure, and kinetics of deoxygenation. The impact of variations in hydrogen partial pressure on the deoxygenation kinetics was analyzed. Furthermore, the microstructural properties of recycled titanium were characterized and evaluated. This study guides advancements in recycling processes within the titanium industry and contributes insights into the improvement in sustainable material practices.

## 2 Experimental Procedures

### 2.1 Hydrogen Plasma Arc Melting of Titanium Alloy Scrap

**Figure 1:**
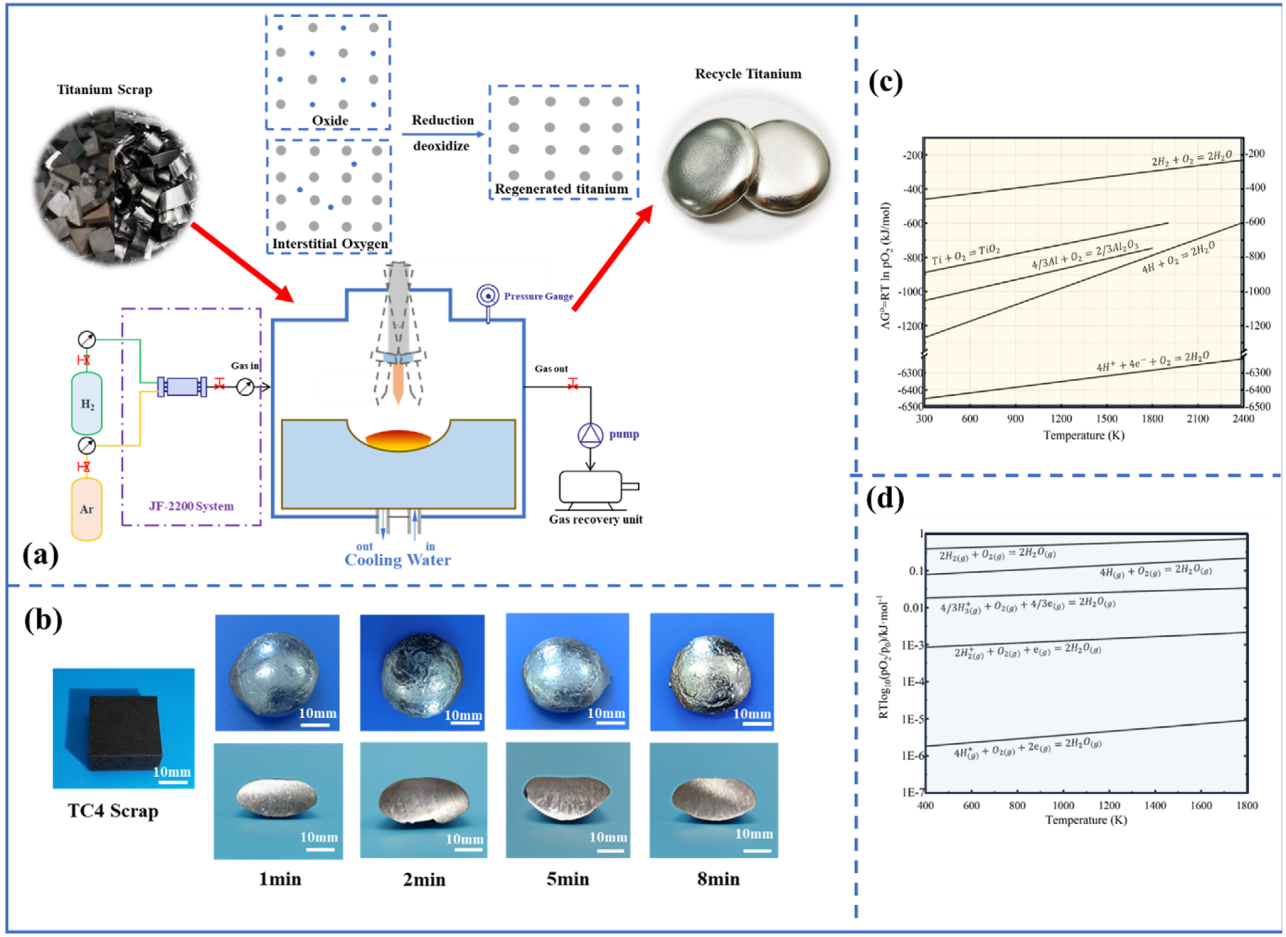
a) Schematic diagram of the recycling of titanium alloy scrap using hydrogen plasma arc melting. b) Macroscopic morphology and cross‐section of the scrap melted for different durations. c) Ellingham diagram for different oxides.^[^ ^23^ ^]^ d) ΔG_0_–T curves for the H_2_O generated from different chemically active hydrogen species.^[^ ^41^ ^]^

The materials used were derived by machining titanium alloy scrap generated during the manufacture of aerospace titanium alloy components. Prior to use, the scrap underwent acid pickling to remove surface contaminants such as oils. A uniformly shaped block of titanium alloy scrap was selected to facilitate characterization. However, the applied technology is adaptable, and specifications regarding the scrap size or shape are not strict. The titanium scrap, with an average weight of 30 g, was placed in an electric arc melting furnace in an atmosphere was composed of 10% hydrogen and 90% argon (total pressure of 5000 Pa). The current and voltage during melting were maintained at 200 A and 40 V, respectively. The scrap was melted one, two, five, or eight times, each with a melting duration of 1 min, to obtain samples from different melting stages. The furnace was recharged with a fresh hydrogen‐argon gas mixture after each one‐minute melting interval to compensate for any hydrogen depletion and ensure that the deoxygenation effect remained consistent. **Figure** **1**a shows a schematic of the recycling of titanium alloy scrap via hydrogen plasma arc melting. Figure 1b shows the macroscopic morphology of the scrap material melted for different durations.

### 2.2 Composition Analysis and Microstructure Characterization

The phases in the titanium scrap were identified using a Panalytical Empyrean X‐ray diffractometer (XRD) in the 2*θ* range of 10° to 90°. A Quanta 200FEG scanning electron microscope (SEM) equipped with an HKL Nordlys electron backscattered diffraction (EBSD) detector was employed for microstructural analysis. Data were collected at an acceleration voltage of 20 kV, with a specimen tilt angle of 70° and a step size of 0.2 µm. The EBSD data were analyzed using OXFORD AZtecCrystal v2.1. The elemental compositions of the composites were determined using electron probe microanalysis (EPMA, JXA‐8230, Japan). The specimens for XRD and SEM analyses were first ground using silicon carbide (SiC) emery papers of various grits (400, 800, 1200, and 2000), which were then mechanically polished using a velvet metallographic polishing cloth (FYJX01) and a chromium trioxide (Cr₂O₃) suspension. Finally, the samples were etched in a mixture of HF, HNO_3_, and water in a 1:1:8 volume ratio for 10 s to expose the microstructure. For EBSD analysis, specimens were electropolished using a solution of methanol, N‐butanol, and perchloric acid (ratio 60:34:6) for 2 min at −10 °C with a 1 A current to remove polishing‐induced stress. The FEI Talos F200X transmission electron microscope (TEM) equipped with a high‐angle annular dark field detector (HADDF) was used for analysis. The TEM operates at 200 kV with a point resolution of 0.25 nm, and the samples were prepared through ion thinning using a GATAN 695 system. The samples were prepared using a dual‐beam focused ion beam system (FIB, Helios Nanolab 600i, FEI, USA) specifically sharpened for 3D atom probe tomography (3DAPT) to analyze the elemental composition and the spatial elemental distribution within the recycled titanium alloy. Atom probe data were collected using a Local Electrode Atom Probe (LEAP 5000XR) system. Data, including visualization, analysis, and 3D atomic reconstruction, were handled using version 6.1 of the AP Suite software.

### 2.3 Tensile Performance Test

Tensile tests at room temperature were conducted with an Instron‐5569 universal testing machine, maintaining a steady crosshead velocity of 1.08 mm min^−1^, equating an estimated strain rate of 1 × 10^−3^ s^−1^. The specimens for these tensile tests were extracted from the ingot's median region, with cross‐sectional dimensions of 6 by 2 mm and a gauge length of 18 mm. The surfaces of all specimens were ground and polished to eliminate any deformations introduced during the wire cutting stage to increase the reliability of the data. The data presented in each curve were derived from the average of three tests.

### 2.4 Temperature Field Simulation

COMSOL Multiphysics finite‐element simulation software was used to model the heating effects of an electric arc plasma on a melt pool under different atmospheric conditions. In this model, the thermal plasma was assumed to partially satisfy the local thermodynamic equilibrium (LTE) conditions. The magnetohydrodynamics (MHD) equations were employed for modeling, and the “equilibrium discharge, in‐plane currents” interface was used to simulate the plasma generated in a direct‐current arc. The tungsten cathode was positioned 5 mm above the Ti‐6Al‐4 V metal in the melt pool with a steady current of 80 A.

## 3 Results and Discussion

### 3.1 Thermodynamic Analysis of Deoxygenation

We analyzed the deoxygenation thermodynamics by first determining the state of the reactants. The hydrogen gas in the chamber dissociates into the plasma region when the plasma arc is activated, resulting in a complex array of dissociation products. The plasma arc region contains molecular hydrogen (H_2_), atomic hydrogen (H), various hydrogen ions (H^+^, H_2_^+^, and H_3_^+^), and vibrationally activated forms (H_2_*), whereas oxygen is primarily present in the form of metal oxides and interstitial oxygen. Therefore, numerous deoxygenation reactions occur during melting.

To simplify the analysis, the reaction between hydrogen gas and titanium oxides can be written as:

(1) $$TiO_{x}+xH_{2}=Ti+xH_{2}O\left(g\right)\Delta G_{1}>0$$

where *TiO_x_* represents titanium oxides in various valence states; Δ*G*_1_ is the Gibbs free energy change of the reaction in Equation 1.

The excitation reaction of hydrogen gas in the plasma can be expressed as:

(2) $$H_{2}=Hydrogen\;Plasma\left(2H|2H^{+}|2H_{2}^{+}\left|2H_{3}^{+}\right|H_{2}^{*}\right)\Delta G_{2}\gg 0$$

The Gibbs free energy change in reaction (2), ΔG_2_, is much larger than zero. By coupling the reactions in Equations 1 and 2, we obtain Equation 3:

(3) $$\begin{array}{ccc} & & TiO_{x}+x\;Hydrogen\;Plasma\left(2H|2H^{+}|2H_{2}^{+}\left|2H_{3}^{+}\right|H_{2}^{*}\right)\\ & & \;=Ti+xH_{2}O\left(g\right)\\ & & \Delta G_{3}=\Delta G_{1}-x\Delta G_{2}<0 \end{array}$$

The spontaneity of a reaction is governed by the change in the Gibbs free energy under specific conditions, with a negative value indicating a spontaneous process. Thus, the reduction of oxides by the hydrogen plasma arc can be inferred as proceeding spontaneously, even at relatively low temperatures. Furthermore, the Gibbs free energy further decreases as the temperature increases, increasing the thermodynamic favorability of the reaction. These findings show that the reduction of oxides in Ti alloys via hydrogen plasma arc melting is thermodynamically feasible.

A more detailed thermodynamic assessment was performed using the Ellingham diagram shown in Figure 1c, which illustrates the Gibbs free energy of various reactions as a function of temperature.^[^ ^24^ ^]^ Molecular hydrogen cannot reduce titanium oxides within the temperature range shown in the diagram according to the thermodynamic principles. However, atomic hydrogen (H) and hydrogen ions (H⁺) have the capacity to facilitate this reduction, with the theoretical reduction potential of H⁺ being several times larger than that of atomic hydrogen. These findings highlight the thermodynamic advantages of this technique.

The interstitial oxygen removal reaction was thermodynamically analyzed by calculating the Gibbs free energy changes for the reactions of molecular hydrogen, atomic hydrogen, and hydrogen ions with oxygen to form H_2_O. The results in Figure 1d demonstrate that the hydrogen species present in the hydrogen plasma arc is thermodynamically suitable for removing interstitial oxygen.^[^ ^41^ ^]^ The deoxygenation efficiency, ranked from strongest to weakest, follows the order H⁺ > H_2_⁺ > H_3_⁺ > H > H_2_.

### 3.2 Microstructural Evolution During Plasma Arc Melting

**Figure 2:**
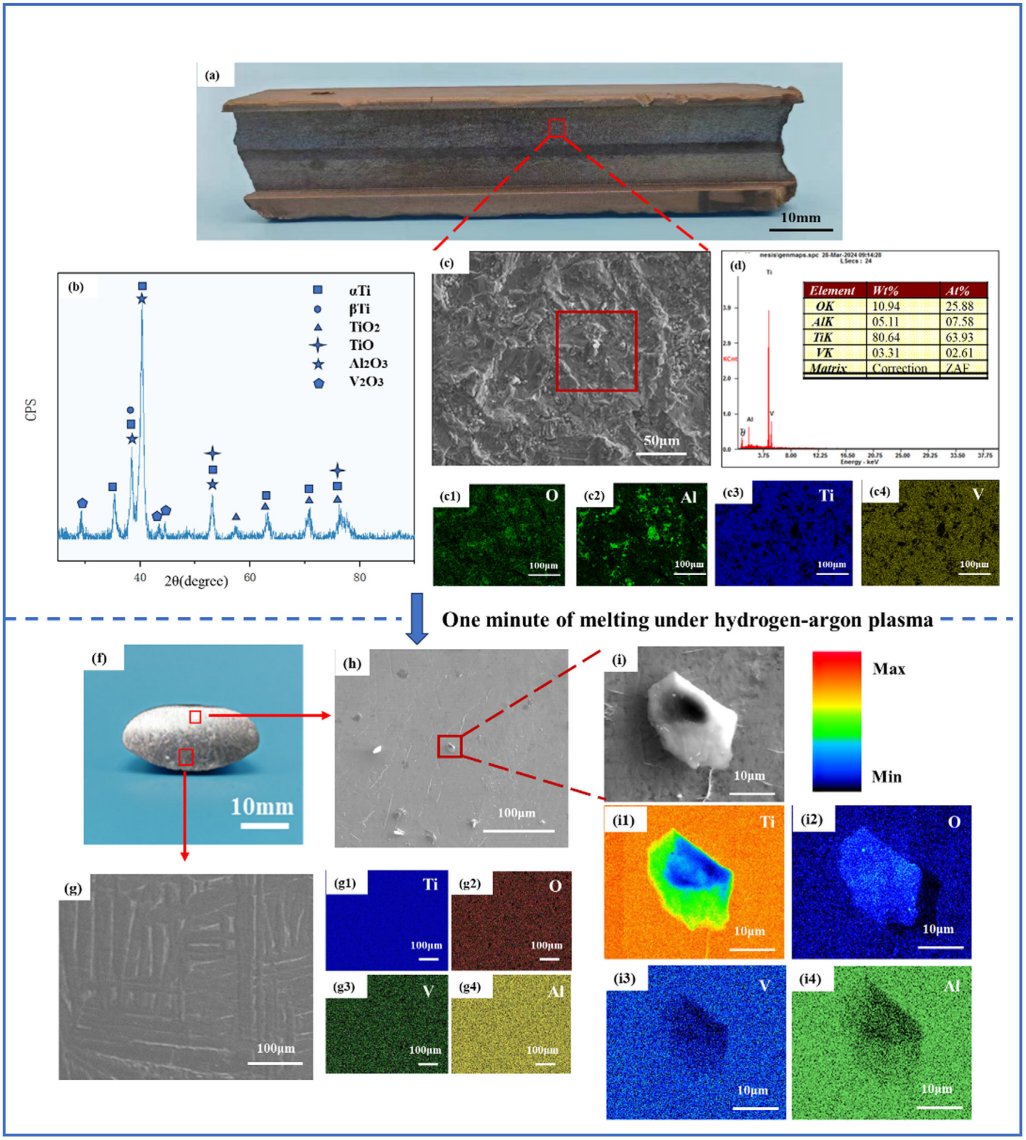
a) Original macroscopic appearance of the titanium allow scrap. b) XRD image of the scrap surface. c) SEM image of the area within the red box in (a). d) The results of elemental content analysis within the area demarcated by the red dashed line in (c). c1–c4) The elemental distribution maps corresponding to (c). f) Cross‐section of the scrap melted for 1 minute. g) Microstructural image of the bottom of the ingot. (g1–g4) The elemental distribution maps corresponding to (g). h) Undissolved oxide particles at the top of the ingot. i) SEM image of the area within the red box in (h). i1–i4) The elemental distribution maps corresponding to (i).

The surface composition and microstructural features of the scrap were characterized to further understand the deoxygenation mechanism, with the results shown in **Figure** **2**. Figure 2a depicts the original morphology of the scrap, with the scrap specimen illustrated in Figure 1b being sourced from this material. The microstructural composition in the region demarcated by the red box was further analyzed. The SEM image in the red‐boxed area reveals the rough surface texture of the scrap in Figure 2c. The XRD pattern in Figure 2b indicates the presence of various metal oxides in this region. This finding is confirmed by the elemental distribution maps in Figure 2(c1–c4). The various oxides formed due to the high temperatures generated during processing, which facilitated complex reactions between the alloy and atmospheric oxygen. Figure 2d shows the results of the elemental analysis of the area within the dashed lines in Figure 2c, which indicate that the oxygen content in this specific region was 25.88%. The contamination of titanium alloy scrap predominantly localized on the surface, as shown in Figure S1 (Supporting Information).

The scrap from various stages of deoxidation were sequentially observed. The surfaces of the samples exhibited a metallic luster without any notable oxidation after one minute of hydrogen plasma arc melting, as shown in Figures 1b and 2f. Figure 2g corroborates this observation, indicating the presence of only the αTi and βTi phases, which corresponds to the XRD results shown in Figure S1 (Supporting Information). Furthermore, the elemental distribution maps in Figure 2(g1–g4) indicate that oxygen remained dissolved in the titanium alloy scrap ingot in the form of interstitial atoms. However, we observed some particulate phases that were not completely dissolved or reduced at the top of the ingot (corresponding to the top of the melt pool), as shown in Figure 2h,i. These particles were further observed. The color ruler represents the variation in element concentration. Figure 2(i1–i4) indicate higher concentrations of oxygen and titanium in this location, suggesting that the particles were likely some forms of titanium oxide compound.

We preliminarily analyzed the deoxygenation process within the first minute of the recycling of the titanium alloy scrap by combining these observations. Hydrogen plasma engages in reductive reactions with the oxides present in the scrap, facilitating deoxygenation. However, a hydrogen plasma arc cannot reduce all the valence states of titanium oxides.^[^ ^42^, ^43^, ^44^ ^]^ These unreduced oxides initially dissolve in the melt pool, and subsequently, this “dissolved oxygen” is removed by the hydrogen plasma. The removal of “dissolved oxygen” is not a reduction reaction in the chemical sense. Therefore, the term “deoxygenation” is consistently used throughout this paper to describe this process. The samples that underwent deoxygenation at intervals of 2, 5, and 8 min were further analyzed (Figure S2, Supporting Information).

**Figure 3:**
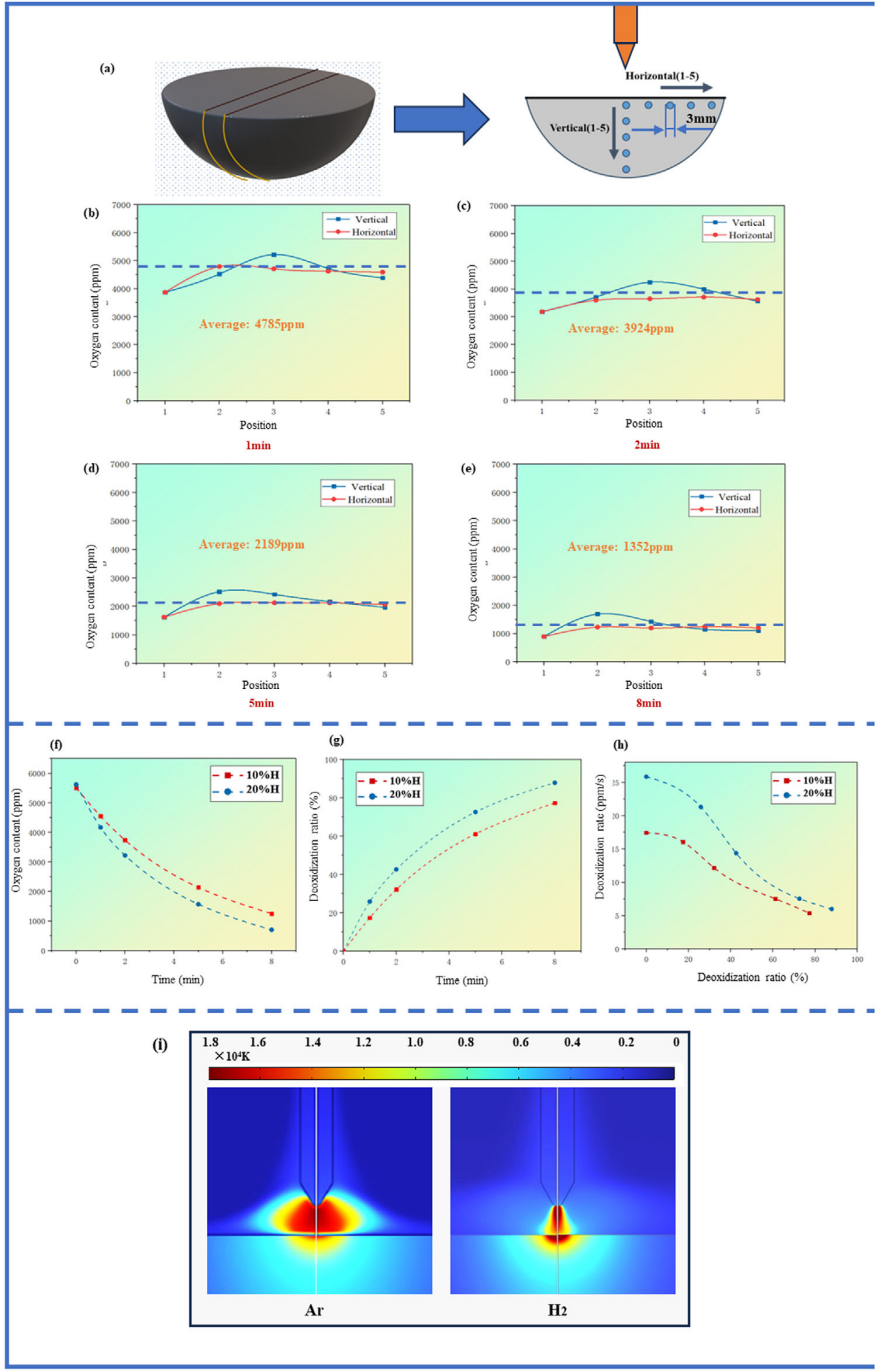
a) Schematic diagram of sampling conducted to measure oxygen content. b–e) Oxygen content of samples that underwent deoxygenation for durations of 1, 2, 5, or 8 min. f) Change in oxygen content with melting duration. g) Change in deoxygenation ratio over time, h) Change in deoxygenation rate as a function of deoxygenation ratio. i) Temperature field models of plasma and melt under different atmospheric conditions.

We quantitatively measured the oxygen content of the ingot at different stages to further evaluate the effectiveness of hydrogen plasma arc melting in deoxygenating the titanium alloy scrap. Small cylindrical samples with a diameter of 3 mm and a height of 3 mm were used to measure the oxygen content. Measurements were recorded at five evenly distributed points in both the horizontal and vertical directions. We conducted measurements on the scrap at evenly distributed points along the midline cross‐section of the material and calculated the average value to ensure accuracy. The samples were uniformly distributed in their respective directions, as shown in **Figure** **3**a. The oxygen content of the ingot was determined as the weighted average of all sample measurements. Figure 3b–e shows the oxygen content distribution curves for the titanium alloy scrap ingots after 1, 2, 5, and 8 min of exposure to hydrogen plasma arc melting, respectively. The dashed lines indicate the average oxygen content. These data, show that the hydrogen plasma arc recycling method used effectively reduced the oxygen content. The oxygen content in the scrap reduced to ≈1352 ppm after being subjected to the plasma arc for eight minutes, which complies with the oxygen specifications for nearly all titanium alloy industrial applications. This indicates that the proposed method has substantial potential for use in practical applications.

Another observation was that oxygen was not uniformly distributed within the ingot. Vertically, the oxygen content initially increased and then decreased from the top down, with the highest oxygen concentration occurring around the middle in the vertical direction. This pattern can be explained by the central top of the ingot, which was directly exposed to the plasma arc, serving as the deoxygenation reaction zone and thus initially containing lower oxygen levels than the other areas. The melt gradually cooled until solidifying into an ingot as the melting ceased. In molten titanium alloys, the solubility of oxygen increases as the temperature decreases.^[^ ^34^ ^]^ The bottom of the melt pool first cooled during solidification (in direct contact with the crucible). This cooling increased the oxygen solubility at the base of the melt pool, generating a force driving oxygen to diffuse downward. Simultaneously, the melt pool solidified. Note that the diffusion of oxygen is hindered as titanium alloy transitions from the liquid to the solid state. Therefore, oxygen enrichment occurred at the solid–liquid interface along the vertical direction.

Horizontally, this phenomenon was less pronounced. Except for deoxygenation reaction zone, which had a lower oxygen content, the oxygen levels in the other locations remained relatively uniform. This phenomenon occurred because the upper surface of the melt pool interfaced with the protective atmosphere of the furnace, which accelerated cooling. The time was insufficient for the diffusion of oxygen prior to the titanium alloy melting and transitioning to a solid state.

### 3.3 Deoxygenation Kinetics Analysis

In the initial part of our study, titanium alloy scrap was recycled through plasma‐arc melting under a 90% argon and hydrogen 10% atmosphere. This approach efficiently reuses material and has a substantial smaller carbon footprint compared with the conventional metal production processes. These benefits underscore the potential of this method to contribute to sustainable metallurgical practices. However, our understanding of process dynamics under different hydrogen partial pressures remained incomplete. We thus extended our experiments by conducting reductions under 20% hydrogen partial pressure to further understand into the effects of hydrogen partial pressure on deoxygenation kinetics. We made this adjustment aimed to provide additional understanding of the kinetic behaviors and mechanisms that occur during the recycling process, refining our knowledge of the dynamics of the process. The results can be used to optimize recycling protocols that maximize both environmental and economic benefits.

The deoxygenation kinetics of the recycling of titanium alloy scrap using hydrogen plasma are shown in Figure 3(f–h). With the same initial oxygen content, more oxygen was removed per unit time with a hydrogen partial pressure of 20%, as shown in Figure 3(f,g). It is worth mentioned that the deoxidation ratio can be calculated using the formula:

(4) $$\begin{array}{ccc} & & Deoxidation\;ratio\;\left(\%\right)\\ & & \;=(1-current\;oxygen\;content/initial\;oxygen\;content)\times 100\;(\%) \end{array}$$

The oxygen removal efficiency was higher at a 20% hydrogen partial pressure because the reactivity of hydrogen with oxygen increased at higher concentrations. Hydrogen acts as a strong reducing agent: hydrogen availability and interactions with oxygen in the alloy increase at higher partial pressures, accelerating the reaction kinetics, leading to the more rapid removal of oxygen from the metal. A higher hydrogen concentration increases the number of reactive sites and the force driving the deoxygenation reaction, thus increasing the efficiency of oxygen scavenging within the same unit of time. Figure 3h shows that increasing the hydrogen partial pressure to 20% markedly boosted the reaction rate relative to that at a 10% hydrogen partial pressure. However, this enhancement in the reaction rate was not linearly related to the hydrogen partial pressure. The kinetics of the deoxygenation process are governed by variables other than the reactant concentration, including the hydrogen diffusion rate and the available reactive surface area on the titanium alloy.

Changes in the hydrogen partial pressure also affect the effective reaction area available for deoxygenation. We simulated the temperature fields of the plasma arc and melt under pure argon and hydrogen atmospheres owing to the complex physicochemical parameters of hydrogen–argon mixtures, with the results shown in Figure 3i. The plasma arc in the pure hydrogen atmosphere had a higher energy density but smaller arc irradiation area than the pure argon atmosphere.

The higher energy density and smaller arc irradiation area in the pure hydrogen environment can be attributed to the physical and electrochemical properties of hydrogen. First, as a light element, hydrogen has a smaller atomic diameter, which allows for a higher ionization rate and the denser clustering of atoms in the plasma state. This results in a higher energy density, which considerably elevates the temperature of the plasma arc, thereby enhancing the localized heating of the melt. Second, hydrogen has a higher thermal conductivity than argon, enabling more effective heat transfer. However, hydrogen rapidly disperses from the high‐temperature zone to the surrounding environment owing to its high diffusion rate, leading to a relatively small effective area for the high‐energy‐density plasma arc. This rapid thermal diffusion helps create a plasma arc that is intensely hot but limited in the affected area. Therefore, the deoxygenation rate depends on the balance between the hydrogen partial pressure and the arc irradiation area.

### 3.4 Microstructure and Mechanical Properties of Recycled Titanium Alloy

The oxygen content of titanium alloy scrap subjected to an eight‐minute deoxygenation process under 10% hydrogen partial pressure using a hydrogen plasma arc meets the industrial standards for Ti‐6Al‐4 V, classifying it as recycled titanium alloy. We further characterized the microstructure and mechanical properties of the recycled titanium alloy to validate its suitability for industrial use.

**Figure 4:**
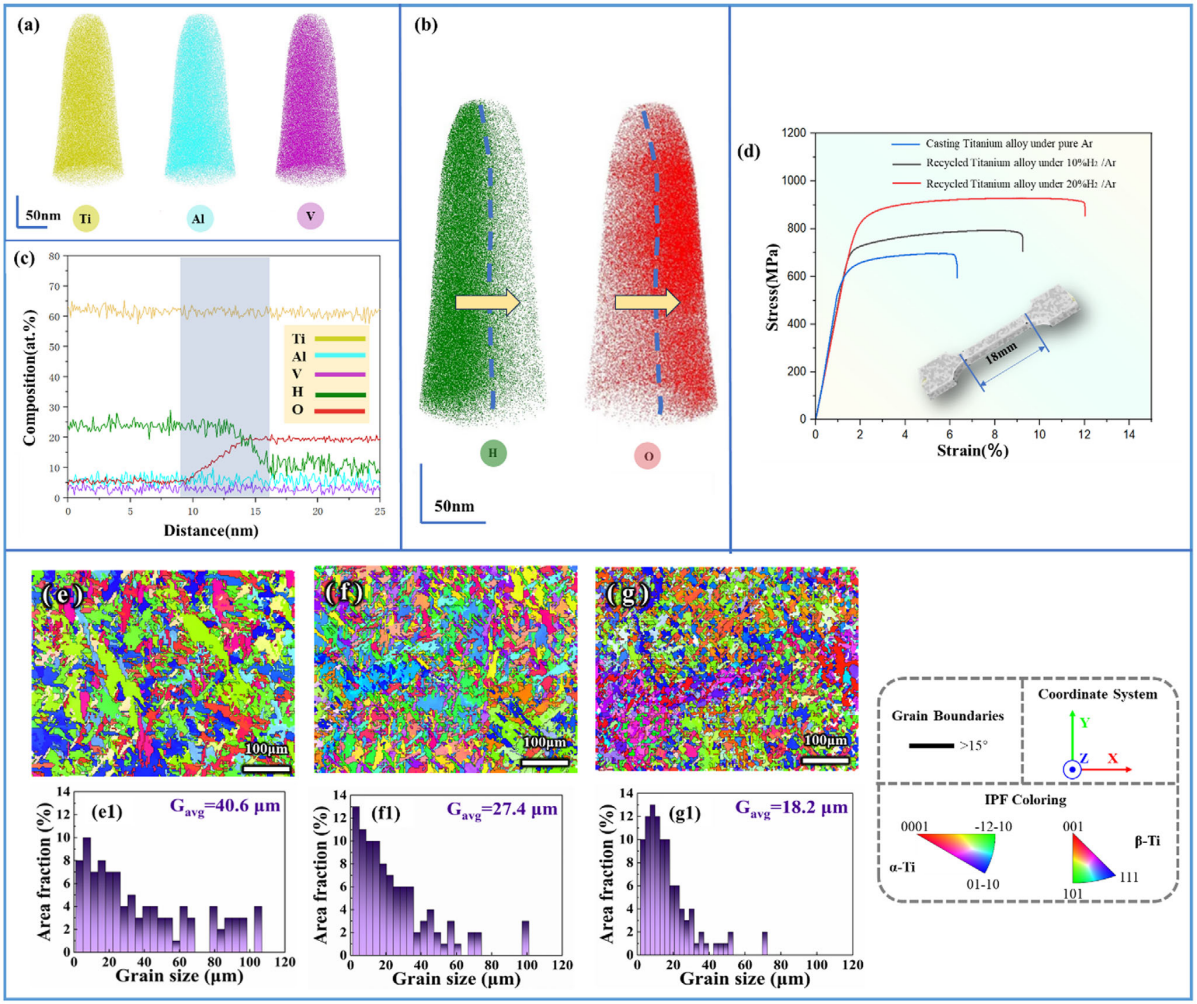
a,b) 3D reconstruction of atom maps for Ti, Al, V, H, and O showing interfacial region of recycled titanium alloys. c) Elemental composition along the direction of the arrow in (b). d) Tensile engineering stress–strain curves of cast and recycled titanium alloys. e–g) IPF Image of casting titanium alloys under pure Ar, 10%H_2_/Ar, and 20%H_2_/Ar. e1–f1) α grain sizes of (e,f).

The distribution of elements, particularly oxygen and hydrogen, within the recycled titanium alloy was characterized using APT, with the results shown in **Figure** **4**. The test samples were specifically selected from the interface region between the α and β phases to highlight the characteristics of the elemental distribution. As shown in Figure 4a, Ti, Al, and V were uniformly distributed throughout the matrix. However, oxygen and hydrogen were distinctly segregated. Oxygen predominantly accumulated in the αTi, whereas hydrogen was primarily found in the βTi, as shown in Figure 4b. Quantitative characterization along the direction indicated by the arrow in Figure 4b revealed variations in the elemental content, as shown in Figure 4c. The changes in the elemental concentrations corresponded to the phenomena observed in the microstructural analysis, where hydrogen and oxygen were enriched in the β and α phases, respectively. This distribution pattern can be attributed to the differences in the solubility and phase stabilization characteristics. The solubility of oxygen and hydrogen differ in the α and β phases of titanium alloys. Oxygen solubility is higher in the α phase, which is hexagonal close‐packed, due to its ability to fit well into the smaller interstitial sites in this structure. Oxygen solubility in the α phase is ≈16 times larger than that in the β phase. Furthermore, the presence of these elements influences the stability of each phase within the alloy. Oxygen hardens and enhances the tensile strength of the α phase, resulting in stabilization, whereas hydrogen functions as a stabilizer for the β phase, potentially inducing phase transformations under specific conditions. This result is consistent with those of the previous studies.^[^ ^36^, ^39^, ^45^, ^46^ ^]^

The mechanical properties of titanium alloys, given their use as structural materials, must be evaluated. Therefore, we conducted tensile tests on the recycled titanium alloy and compared its performance with that of conventionally cast titanium alloy, with the results illustrated in Figure 4d. The recycled titanium alloys exhibited increases in both strength and elongation compared with the cast titanium alloys. Specifically, the tensile strength increased by 24.6% from 696 to 924 MPa. The elongation increased from 6.31% to 12.02%, an increase of ≈90.5%. To further characterize the microstructure and phase constituents, the recycled titanium alloy was analyzed using TEM. Figure S3a (Supporting Information) shows the bright‐field image of the titanium alloy recycled under 10% H₂/Ar. The image reveals the presence of only two phases distributed alternately. Selected area electron diffraction (SAED) patterns in Figure S3(a1,a2) (Supporting Information) confirm that these two phases are α‐Ti and β‐Ti, which are typical for Ti‐6Al‐4V. Figure S3b (Supporting Information) presents the bright‐field image of the titanium alloy recycled under 20% H₂/Ar. The phase constituents are consistent with those in Figure S3a (Supporting Information), containing only α‐Ti and β‐Ti, with no evidence of hydride phases or other phases such as α', as indicated by the SAED patterns in Figure S3(b1,b2) (Supporting Information). However, the grain size appears to have changed. To quantify this further, EBSD was employed. Figure 4(e,f) shows the inverse pole figure (IPF) maps, revealing that the grains of the recycled titanium alloy obtained using our technique were more refined and more randomly oriented. The grain size distribution was analyzed and the average grain size was calculated to provide further quantitative insight into these results, as shown in Figure 4(e,f). The recycled titanium alloy melted in a 20%H₂/Ar atmosphere had an average grain size of ≈18.2 µm, which was 44.8% of that of the as‐cast titanium alloy (40.6 µm) melted in a pure Ar atmosphere. Therefore, the increases observed in the strength and ductility of the recycled titanium alloy can primarily be attributed to microstructural refinement and an increase in the softer, more ductile β phase within the alloy. Hydrogen plays a role in the solidification of recycled titanium alloys by adsorbing at the forefront of the solid–liquid interface, which impedes grain growth. The addition of hydrogen resulted in the formation of more nucleation sites. Consequently, the microstructure of the recycled titanium alloy was refined, leading to increases in both the strength and toughness. Additionally, the increase in the amount of the β phase (an increase of 52.3% under 20%H_2_/Ar, as shown in Table S1, Supporting Information), which is known for increased malleability compared with that of the α phase, contributes to the alloy's overall toughness and flexibility. This phase distribution modified the mechanical behavior by accommodating more deformation before failure, thus increasing the elongation of the recycled titanium alloy. The mechanisms through which the recycled titanium alloys are strengthened and toughened deserve further investigation.

## 4 Conclusion

1. The hydrogen plasma arc offers thermodynamic advantages over traditional methods for removing oxygen from a melt.
2. The deoxygenation of titanium alloy scrap achieved using hydrogen plasma arc primarily involves the reduction and dissolution of oxides, as well as the removal of dissolved oxygen.
3. The deoxygenation kinetics achieved with a hydrogen plasma arc are determined by the balance between the hydrogen partial pressure and the arc irradiation area.
4. In the recycled titanium alloy, oxygen tends to be enriched in αTi whereas hydrogen tends to be enriched in βTi.
5. The strength and elongation of the recycled titanium alloy are higher than those of cast titanium alloy.

## Conflict of Interest

The authors declare no conflict of interest.

## Supporting information



Supporting Information

## Data Availability

The data that support the findings of this study are available from the corresponding author upon reasonable request.
