# Supplementary material for: Environmentally Friendly and Simple Recycling of Titanium Alloy Scrap via Deoxygenation with Hybrid Hydrogen Plasma Arc
Source: Adv Sci (Weinh). 2025 Jan 21;12(10):2414747. doi: 10.1002/advs.202414747 (PMC11904995; doi:10.1002/advs.202414747)
Supplement: Supplementary file 1 — Supporting Information [file ADVS-12-2414747-s001.docx]

***Supplementary material***

**Environmentally Friendly and Simple Recycling of Titanium Alloy Scrap via Deoxygenation with Hybrid Hydrogen Plasma Arc**

*Botao Jiang^1,2,3^, Liang Wang^1,2,3*^, Guotao Zhou^1^, Xu Liu^1^, Hongyu Lu^1^, Guoqiang Zhu^1^, Jiaxin Du^1^, Chunzhi Zhao^1^, Baoxian Su^1,2,3*^, Binbin Wang^1^, Ruirun Chen^1,3^, Yanqing Su^1,2,3*^*

*1 School of Materials Science and Engineering, Harbin Institute of Technology, Harbin 150001, China*

*2 Zhengzhou Research Institute, Harbin Institute of Technology, Zhengzhou 450000, China*

*3 National Key Laboratory for Precision Hot Processing of Metals, School of Materials Science and Engineering, Harbin Institute of Technology, Harbin 150001, China*

**submitted to**

**Advanced Science**

**January 2025**

* Correspondence and requests for data and materials should be addressed to Liang. Wang (email: wliang1227@hit.edu.cn), Baoxian. Su (email: subaoxian@hit.edu.cn) and Yanqing. Su (email: suyq@hit.edu.cn)


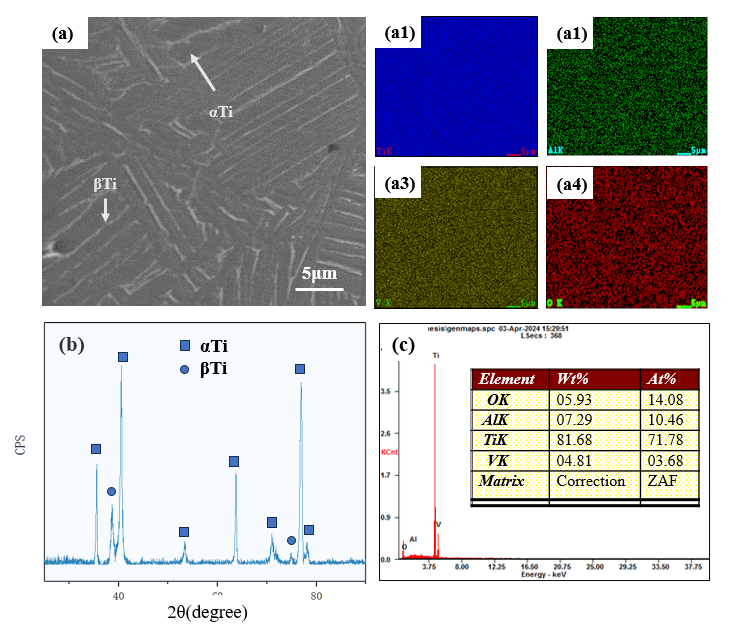


Fig. S1(a) Microstructural of the Interior of Titanium Alloy Scrap. (a1-a4) The elemental distribution maps corresponding to (a). (b) XRD image of the Interior of Titanium Alloy Scrap. (c) The analysis of elemental content in (a)

Fig. S1 presents the characterization of the scrap's internal microstructure. From Fig. S3(a), it is evident that there are no oxides present within the scrap. The XRD results shown in Fig. S1(b) also indicate that only the αTi and βTi phases exist. As titanium alloy scrap predominantly originates from machining processes, the attendant contamination and oxidation are largely localized to the scrap's surface. Nonetheless, this does not necessarily imply that the oxygen content is within acceptable levels. A more detailed characterization of the composition and elemental distribution within the interior of the scrap is provided in Fig. S1(a1-a4) and (c), revealing that while oxides have not formed, oxygen exists as interstitial atoms within the titanium alloy scrap and is uniformly distributed throughout.


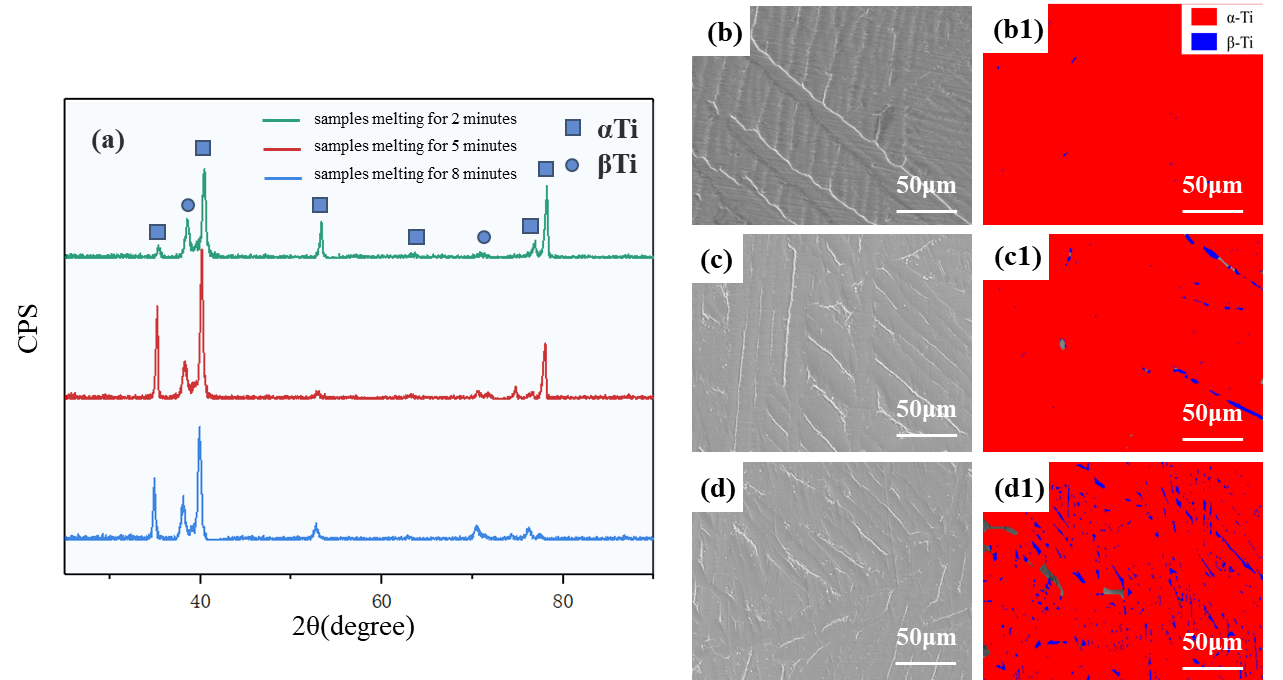


Fig. S2(a) XRD analyses of samples that had undergone deoxygenation for intervals of 2, 5, and 8 minutes. (b-d) SEM images of samples that had undergone deoxygenation for intervals of 2, 5, and 8 minutes. (b1-d1) EBSD phase color map of samples that had undergone deoxygenation for intervals of 2, 5, and 8 minutes.

XRD analyses presented in Fig. S2(a) consistently show the presence of only αTi and βTi phases across these stages. The SEM images from Figs. S2(b-d) confirm the absence of any undissolved oxide particles within the ingots, indicating that reduction reactions cease and deoxygenation predominantly occurs through the elimination of dissolved oxygen. Figs. S2(b1-d1) display the phase color maps of the recycled titanium alloy from different stages, with αTi indicated by red and βTi by blue. According to the EBSD results, an interesting phenomenon observed is the gradual increase of the β phase in the titanium alloy. This is due to the rising content of hydrogen dissolved in the alloy as the melting duration increases. Hydrogen, acting as a β-stabilizing element, promotes the retention of more βTi in the material.

Table S1. β phase content of titanium alloys under different atmospheres.

| Types of atmospheres | Ar | 10%H_2_/Ar | 20%H_2_/Ar |
| --- | --- | --- | --- |
| β phase content (%) | 4.4 | 5.9 | 6.5 |

*
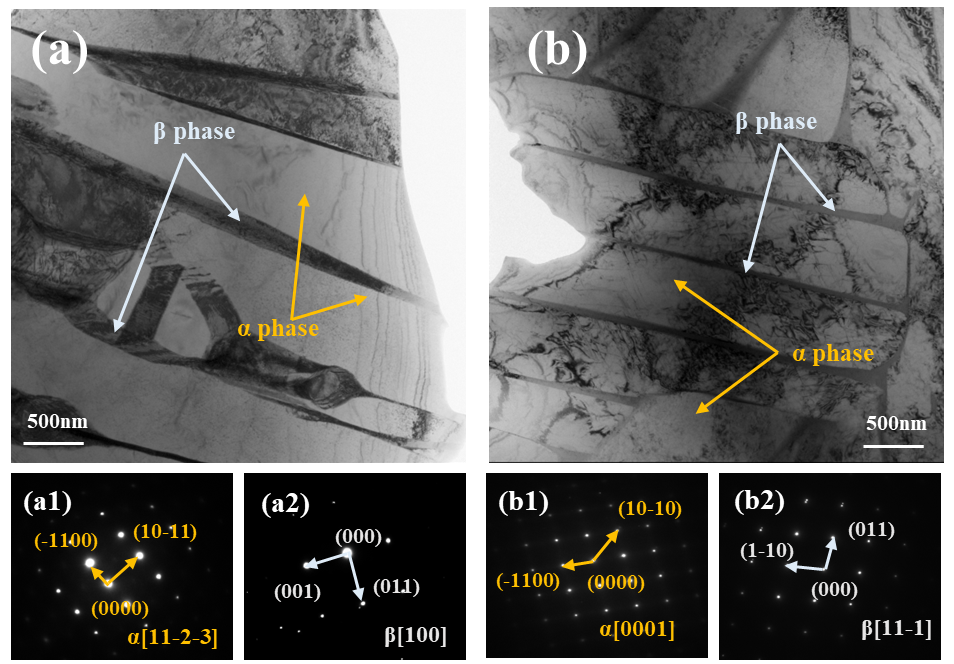
*

**Figure. S3** Typical bright-field TEM images and SAED patterns: (a-a2) recycled titanium alloys melt under 10%H2/Ar. (b-b2) recycled titanium alloys melt under 20%H2/Ar.
